# Measuring safety culture in Dutch primary care: psychometric characteristics of the SCOPE-PC questionnaire

**DOI:** 10.1186/1472-6963-13-354

**Published:** 2013-09-17

**Authors:** Natasha J Verbakel, Dorien LM Zwart, Maaike Langelaan, Theo JM Verheij, Cordula Wagner

**Affiliations:** 1 Department of General Practice, Julius Center for Health Sciences and Primary Care, University Medical Center Utrecht, Utrecht, The Netherlands; 2 Nivel, Netherlands Institute for Health Services Research, Utrecht, The Netherlands; 3 Department of Public and Occupational Health, EMGO+ Institute, VU University Medical Center, Amsterdam, The Netherlands

**Keywords:** Primary care, Patient safety, Safety culture, Questionnaire, Psychometric characteristics

## Abstract

**Background:**

Patient safety has been a priority in primary healthcare in the last years. The prevailing culture is seen as an important condition for patient safety in practice and several tools to measure patient safety culture have therefore been developed. Although Dutch primary care consists of different professions, such as general practice, dental care, dietetics, physiotherapy and midwifery, a safety culture questionnaire was only available for general practices. The purpose of this study was to modify and validate this existing questionnaire to a generic questionnaire for all professions in Dutch primary care.

**Methods:**

A validated Dutch questionnaire for general practices was modified to make it usable for all Dutch primary care professions. Subsequently, this questionnaire was administered to a random sample of 2400 practices from eleven primary care professions. The instrument’s factor structure, reliability and validity were examined using confirmatory and explorative factor analyses.

**Results:**

921 questionnaires were returned. Of these, 615 were eligible for factor analysis. The resulting SCOPE-PC questionnaire consisted of seven dimensions: ‘open communication and learning from errors’, ‘handover and teamwork’, ‘adequate procedures and working conditions’, ‘patient safety management’, ‘support and fellowship’, ‘intention to report events’ and ‘organisational learning’ with a total of 41 items. All dimensions had good reliability with Cronbach’s alphas ranging from 0.70 – 0.90, and the questionnaire had a good construct validity.

**Conclusions:**

The SCOPE-PC questionnaire has sound psychometric characteristics for use by the different professions in Dutch primary care to gain insight in their safety culture.

## Background

One of the main focuses in patient safety research is patient safety culture. A supportive patient safety culture is seen as an important condition for patient safety [1]. Patient safety culture refers to values, attitudes, norms, beliefs, practices, policies, and behaviours about safety issues in daily practice. In essence, culture is “the way we do things around here” [2]. In a review, Sammer et al. identified seven subcultures of patient safety culture: leadership, teamwork, evidence based, communication, learning, just, and patient-centred [3]. Gaining insight in the prevailing safety culture is therefore seen as a first pivotal step towards an adequate patient safety system [4]. Various instruments have been developed to measure patient safety culture [2,5-8]. They help to identify weak areas in the perceived safety culture and thus enable designing tailored improvement strategies.

In recent years, increasing attention has been given to patient safety in primary care [9-14]. Primary care is directly accessible and consists of a broad array of professions, e.g. dental care, general practice, physiotherapy, midwifery, speech therapy. Despite this wide range of care, practices have many similarities in organisational structure. As most practices are small, managerial and organisational tasks -including safety improvement- are mostly done by the professionals themselves. Moreover, primary care professionals increasingly work together in broad healthcare centres, collaborating in disease management programmes and consulting one another in managing the care of individual patients.

Because of the increase in collaboration within primary care, developing a generic patient safety culture instrument was desirable. It will enable comparison between different primary care providers and in a later stage of safety management, may generate exchange of learning and improvement strategies. As a tool for patient safety culture already exists in the Netherlands: the SCOPE, it has been developed and validated for general practice only [15]. SCOPE is a Dutch acronym for systematic culture inquiry on patient safety. Other primary care professionals were already familiar with it, therefore, we choose to modify this tool into a generic questionnaire for all professions in primary care: the SCOPE-Primary Care (SCOPE-PC).

## Methods

### First adjustment to the questionnaire

The Dutch SCOPE questionnaire for general practices, is a modification of the Dutch Hospital Survey on Patient Safety (HSOPS) [15,16]. This original SCOPE for general practice consists of eight dimensions:

1. Handover and teamwork (8 items)

2. Support and fellowship (5 items)

3. Communication openness (6 items)

4. Feedback about and learning from error (6 items)

5. Intention to report events (3 items)

6. Adequate procedures and adequate staffing (7 items)

7. Overall perceptions of patient safety management (4 items)

8. Expectations and actions of managers (4 items).

We made adjustments to this SCOPE through an iterative process. First, the research team revised the terminology of the questionnaire. Secondly, professionals from all primary care professions assessed the questionnaire individually clarity and applicability to their own setting. In total 27 professionals (1 midwife, 4 pharmacists, 1 physician, 2 dieticians, 2 physician assistants, 2 physiotherapists, 2 skin therapists, 2 general practitioners, 1 speech therapist, 2 dental hygienists, 2 exercise therapists, 2, dentists, 2 dentist assistants, 1 occupational therapist, 1 general practice nurse and 1 nurse working in an anticoagulation clinic) gave feedback by e-mail. Lastly, the research team reached consensus on the version to be used for the further validation process.

Adjustments were limited to a few changes of terminology, for example ‘general practitioner’ was changed to ‘professional’ and ‘physician assistant’ was changed to ‘support staff’. None of the original patient safety culture items were deleted. Three questions were added for routing purposes, where if this question prompted a negative answer the respondent was not shown the other questions regarding this topic. One question was added: ‘*Are incident reports discussed in meetings on a structural basis? Structural means that it is a permanent feature on the agenda’*.

Besides patient safety culture questions the existing questions about patient safety characteristics of the practice were included: whether or not events were discussed in an informal way, the frequency of event reports filled in in the last 12 months and a patient safety grade for the total practice (answer categories: failing, poor, acceptable, good, excellent).

The final questionnaire consisted of 43 patient safety culture items. Items had to be answered using a five-point Likert scale ranging from strongly disagree (1) to strongly agree (5) or never (1) to always (5). On request of the individual professionals we added the option ‘not applicable’ to the questions about the practice organisation and collaboration. Background questions addressed demographics and work-related information, such as how long and in which profession the respondent had been working in this practice.

### Data collection and respondents

Data collection for validation of the questionnaire took place from March until May 2011. An online system managed by the Dutch Practices Accreditation Organisation was used for collection and storage of the data [17].

Eleven primary care professions participated: dental care, dental hygienist care, dietetics, exercise therapy, physiotherapy, occupational therapy, midwifery, anticoagulation clinics, general practice, skin therapy and speech therapy. A random sample of 200 members was drawn from the national databases of each professional association. These members were asked to participate and to invite colleagues from their own practice too. The key to sign in to the digital questionnaire was included in the invitation. It was emphasized that the questionnaire was to be filled out individually. In addition, practices were promised a feedback report regarding the patient safety culture of their practice.

The selection process differed for one of the professions: the physiotherapists were invited directly by their professional association. Because of this extra step, a lower response rate was expected. To anticipate on this, the sample for physiotherapists was doubled to 400. Once enrolled, the inclusion and the following steps were the same as for the other professions.

All practices received a first invitation followed by two reminders with an interval of three weeks to all the contact persons. Invitations and reminders were preferably sent by e-mail but if not available by post.

## Analyses

### Preliminary analyses

As culture is a feature of a group, single-handed practices without employees were excluded from analyses. In addition, as it takes time to absorb the culture of an organisation, we excluded respondents with less than half a year experience in their current practice. Further, respondents with more than five missing values on the patient safety culture items were excluded. The answer category ‘not applicable’ was not counted as missing. Items that were negatively worded were recoded so that high scores always reflect a positive response. Subsequently, distributions of variables were examined to assess response variability and missing data. Inter-item correlations were studied, as well as Bartlett’s test of sphericity and the Kaiser-Meyer-Olkin Measure of Sampling Adequacy (KMO) were performed to see whether a factor analysis could be performed. When Bartlett’s test is significant (p<0.001) it indicates that the data are appropriate for factor analysis. For KMO a value near 1 indicates that patterns of correlations are relatively compact and factor analysis should yield distinct and reliable factors [18]. Regarding the rule of thumb of 10 respondents per patient safety culture item, at least 430 completed questionnaires were needed [19].

### Factor analyses

As we built on an existing questionnaire, a confirmatory factor analysis (CFA) was performed to investigate whether the structure of the original SCOPE for general practices could be confirmed for these data (for explanation of the CFA see Additional file 1). The χ^2^ and RMSEA were used as parameters for goodness of fit. A non-significant χ^2^ means that the discrepancies between the hypothesized model and the empirical data are negligibly small and thus indicate a good fit. The RMSEA measures how well the empirical model approaches the theoretical model. A value of <0.05 is considered a close fit of the model, a value of <0.08 fair or a reasonable error of approximation, and values >0.1 are regarded as not acceptable [20,21].

To examine whether a different structure would give a better fit to the data, an explorative factor analysis (principal component analysis, promax rotation) was performed. To determine how many factors should be retained the eigenvalues and the scree plot (see Additional file 2) were examined. Also, the total explained variance was taken into account.

### Reliability

Internal consistency of the factors was measured using Cronbach’s alpha. A Cronbach’s alpha of >.60 indicates that different items measure the same concept [18]. A positive rating for internal consistency is met when Cronbach’s alphas range between .70 and .95 [19]. We also examined the deleted-item reliability coefficients.

### Construct validity

For all respondents, sum scores were calculated by obtaining the mean score of all items within one dimension. One missing value per dimension was allowed. Subsequently, intercorrelations between dimensions were calculated with Pearson correlation coefficients. We expected that the various dimensions would correlate moderately as they cover an aspect of the same construct: patient safety culture. However, the correlations should not exceed .70 because this would mean that the dimensions are too similar and measure the same concept. Furthermore, correlations of the dimensions with the patient safety grade were computed. It was expected that all dimensions would have a positive correlation with the grade.

All Statistical analyses were conducted using SPSS 17.0 and Lisrel 8.8 for the CFA [22].

### Ethics statement

The Medical Research Ethics Committee of the University Medical Center Utrecht concluded that no WMO approval for this study was needed.

## Results

In total, 921 individual questionnaires were returned from 519 practices. 306 questionnaires were excluded for further analysis: 200 from single-handed practices, 11 from respondents with less than half a year experience at the particular practice and 94 with more than 5 missing values, resulting in 615 questionnaires eligible for the study. Bartlett’s test was significant (p<0.001) and the KMO was 0.91 indicating that the data were appropriate for a factor analysis.

Table 1 gives a description of the study population by gender and age and response rate per profession. Overall, the age and gender distribution of the sample was representative for the Dutch professions population, where reference data were available (data not shown). However, in both dental care and general practice females were overrepresented.

**Table 1:** Study population by age and gender and response rate per profession

**Professions**	**n**	**Response rate**	**Age mean (SD)**	**% women**
Exercise therapy practices	35	17.5%	42 (9.7)	97.1
Dental care practices	42	21%	42 (11.8)	67.5
Dental hygiene practices	14	7%	40 (10.1)	100
Dietetic practices	18	9%	41 (10.3)	100
Midwifery practices	123	61.5%	37 (10.3)	96.7
Occupational therapy practices	34	17%	41 (8.5)	93.5
Physiotherapy practices	147	36.8%	40 (12.2)	50.4
Anticoagulation clinics	95	47.5%	49 (9.1)	87.8
General practices	16	8%	48 (10.7)	50
Skin therapy practices	24	12%	35 (11.7)	100
Speech therapy practices	67	33.5%	37 (10)	100
**Total**	**615**	**25.6**%	**41 (11.4)**	**81.7**

### Confirmatory factor analysis

A confirmatory factor analysis showed that the original factor structure did not fit well with the data (χ^2^=2855.56 df=832, p<0.001). The RMSEA was .06 (90% confidence interval: .06-.07). Although some authors consider a value of .06 a fair fit, the cut-off point of <.05 is usually used. Therefore, also an exploratory factor analysis was conducted to examine if a different structure would fit the data better.

### Exploratory factor analysis

Exploratory factor analyses showed the best fit with seven dimensions: (1) open communication and learning from errors, (2) handover and teamwork, (3) adequate procedures and working conditions, (4) patient safety management, (5) support and fellowship, (6) intention to report events and (7) organisational learning. The original dimension ‘*communication openness*’ was divided in two dimensions: ‘*open communication and learning from error*’ and ‘*adequate procedures and working conditions*’. Table 2 provides an overview of the seven dimensions, the number of items with their factor loading, mean score and standard deviation (SD) per dimension.

**Table 2:** Mean scores and factor loadings of the items of the SCOPE-PC questionnaire

**Item**	**Description**	**Mean**	**SD**	**F1**	**F2**	**F3**	**F4**	**F5**	**F6**	**F7**	**α**
**Open communication and learning from error**											.87
C1	We are given feedback about changes put into place based on event reports	3.95	1.27	.84							
C2	Staff will freely speak up if they see something that may negatively affect patient care	4.53	0.65	.59							
C3	We are informed about errors that happen in this practice	4.22	0.88	.86							
C4	Staff feel free to question the decisions or actions of those with more authority	4.08	0.89	.72							
C5	In this practice, we discuss ways to prevent errors from happening again	4.42	0.76	.69							
C7	Professionals discuss errors that occurred with each other	4.30	0.78	.73							
C9	We are given personal feedback about our own event reports	4.09	0.99	.66							
B4n	My supervisor/manager overlooks patient safety problems that happen over and over	3.96	0.81	.40							
**Handover and teamwork**											.87
F1n	Problems often occur in the exchange of information across disciplines in our practice	3.50	1.01		.67						
F2n	The fact that patients are treated by different professionals in our practice is causing problems	4.12	0.71		.77						
F3n	Disciplines in the practice that we co work with do not coordinate well with each other	3.88	0.90		.85						
F4	There is a good exchange of information between professionals in this practice	4.30	0.76		.52						
F5	There is a good exchange of information between supporting staff in this practice	4.21	0.72		.45						
F7n	Things “fall between the cracks” when transferring patients between different disciplines in this practice.	3.89	0.88		.83						
F8n	Important patient care information is often lost because patients see different professionals	4.01	0.85		.81						
**Adequate procedures and working conditions**											.86
A5n	It is just by chance that more serious mistakes don’t happen around here	4.34	0.78			.77					
A7n	We use more agency/temporary staff than is best for patient care	4.40	0.78			.80					
A8n	Staff feel like their mistakes are held against them	4.23	0.80			.54					
A10n	In this practice we work longer hours than is best for patient care	3.89	0.92			.76					
A12n	When an event is reported, it feels like the person is being written up, not the problem	4.06	0.80			.65					
A13n	We work in “crisis mode” trying to do too much, too quickly	3.80	0.95			.59					
A14n	Staff worry that mistakes they make are kept in their personnel file	4.17	0.77			.58					
A15n	We have patient safety problems in this practice	4.39	0.70			.59					
B3n	Whenever pressure builds up, my supervisor/manager wants us to work faster, even if it means taking shortcuts	4.02	0.84			.43					
**Patient safety management**											.86
B1	My supervisor/manager says a good word when he/she sees a job done according to established patient safety procedures	3.32	0.96				.71				
B2	My supervisor/manager seriously considers staff suggestions for improving patient safety	3.96	0.73				.86				
B5	My supervisor/manager provides a work climate that promotes patient safety	3.90	0.73				.96				
B6	The actions of my supervisor/manager show that patient safety is top priority	3.76	0.88				.90				
B7n	My supervisor/manager seems interested in patient safety only after an adverse event happens	4.09	0.74				.43				
**Support and fellowship**											.83
A1	People support one another in this practice	4.56	0.62					.90			
A2	We have enough staff to handle the workload	3.93	0.94					.60			
A3	When a lot of work needs to be done quickly, we work together as a team to get the work done	4.18	0.75					.85			
A4	In this practice, people treat each other with respect	4.51	0.63					.92			
A11	When someone in this practice gets really busy, others help out	4.12	0.74					.79			
**Intention to report events**											.90
D2	When a mistake is made, but is caught and corrected before affecting the patient, how often is this reported?	3.56	1.19						.91		
D3	When a mistake is made, but has no potential to harm the patient, how often is this reported?	3.59	1.14						.93		
D4	When a mistake is made that could harm the patient, but does not, how often is this reported?	4.01	1.04						.90		
**Organisational learning**											.70
A6	We are actively doing things to improve patient safety	3.95	0.82							.62	
A9	Mistakes have led to positive changes here	3.97	0.68							.57	
A16	Our procedures and systems are good at preventing errors from happening	4.00	0.66							.53	
**Deleted items**											
C6n	Staff are afraid to ask questions when something does not seem right										
F6	Disciplines work together well to provide the best care for patients										
**Separate item**											
C8	Professionals discuss errors that occurred with other disciplines	3.55	1.08								

The letter “n” in an item-code means that it concerns an item in negative wording.

Two items did not have a satisfactory loading above .40 on any of the factors and one item loaded on two factors. Two of these items: ‘*Staff are afraid to raise questions if something does not seem right*’ and ‘*Disciplines work together well to provide the best care for patients*’ were deleted from the questionnaire, since the content of these items were covered to a great extent by other items. As the content of the item *‘Professionals discuss errors that occurred with other disciplines’* was not covered by other items, and in view of the expected increase of interdisciplinary collaboration in the near future, it was retained as a separate item. A few items fell under a different dimensions than in the original SCOPE. The answer category ‘not applicable’ was retained for the question ‘*When one area in this practice gets really busy, others help out’* and questions regarding collaboration. The seven dimensions jointly explained 58.9% of variance.

### Reliability and construct validity

The internal consistency was excellent with Cronbach’s alphas ranging from .70 to .90 (see Table 2). Table 3 shows the mean dimension scores with the SD and correlations between the seven dimensions and the patient safety grade. Overall, the correlations between the dimensions were moderate to good. The highest correlations were between ‘*open communication and learning from error’* and ‘*patient safety management’* (r=.61) and between ‘a*dequate procedures and working conditions’* and ‘*handover and teamwork*’ (r=.57). ‘*intention to report events’* did not correlate with other dimensions (r= .11 - .18) except for *‘open communication and learning from error’* (r=.38). The correlation with the patient safety grade showed a similar pattern: for all dimensions there was a moderate to good positive correlation ranging from .34 to .55 except for ‘*intention to report events*’ (r=.21).

**Table 3:** Mean dimension scores, correlation with patient safety grade and intercorrelations of the seven dimensions

**Dimensions**		**n**	**Mean (SD)**	**Patient safety grade**	**1**	**2**	**3**	**4**	**5**	**6**
1	Open communication and learning from error	588	4.22 (0.64)	0.44**						
2	Handover and teamwork	456	3.99 (0.62)	0.43**	0.50**					
3	Adequate procedures and working conditions	457	4.12 (0.54)	0.47**	0.53**	0.57**				
4	Patient safety management	294	3.81 (0.65)	0.55**	0.61**	0.53**	0.52**			
5	Support and fellowship	606	4.26 (0.60)	0.34**	0.40**	0.42**	0.46**	0.39**		
6	Intention to report events	590	3.72 (1.03)	0.21**	0.38**	0.11*	0.15**	0.17**	0.18**	
7	Organisational learning	609	3.97 (0.59)	0.42**	0.41**	0.38**	0.33**	0.49**	0.54**	0.20**

** Correlation is significant at the 0.01 level (2-tailed).

* Correlation is significant at the 0.05 level (2 tailed).

## Discussion

### Main findings

Validation of the SCOPE-PC showed that the questionnaire consisted of seven dimensions, slightly differing from the original SCOPE questionnaire with eight dimensions. The main difference was that the original dimension ‘*communication openness’* in the current study was divided in two dimensions: ‘*open communication and learning from error*’, and ‘*adequate procedures and working conditions’*. Internal consistency and construct validity were good.

### Interpretation of findings

It is interesting to note the absence of a correlation between ‘*intention to report events*’ and all other dimensions but one: ‘*open communication and learning from error*’. The absence of correlation between ‘*intention to report*’ and most other dimensions may be explained by a difference in perspective. The questions about reporting relate to actual steps to be undertaken when an error occurs, they ask about one’s personal intentions: What would you *do* if? In contrast, questions regarding collaboration, support, the notion of abiding and employing the procedures about patient safety relate to how everybody *feels* or thinks of the atmosphere in their practice, and is concerned with how this is at the moment.

Another explanation for the absence of correlation could be the fact that reporting is still very uncommon in primary care. The dimension ‘*intention to report events*’ does therefore not ‘behave’ the way the other dimensions do. Additionally, the fact that *‘open communication and learning from error’* does correlate may indicate that this is an important precondition for reporting. Subsequently, one would expect that the coherence of all dimensions will become stronger as safety management activities become common practice.

The uneven distribution of the response rate in the eleven professions was striking. A partial explanation is that some professionals, like general practitioners, often receive requests to participate in a study and are therefore less likely to respond. Also, some general practitioners had already completed the original questionnaire during their accreditation process. The original questionnaire was already available for them. Furthermore, it is possible that the interest for patient safety differs between primary care professions. Despite these differences we considered all professions as one group; similarities in the targeted patient group, organisational structure and educational level of employees justify this.

### Strengths and limitations

The strength of this questionnaire is that it serves all primary care professions with one generic questionnaire. This facilitates comparison in further research.

This study has some limitations. First, selection bias due to the fact that more innovative practices and practices that are more enthusiastic about the topic were probably more willing to participate, could not be excluded. Because data collection was done through a contact person, we may assume that personal drive and maybe even authority played a role in participation of individuals. However, for a psychometric study this is less important since the focus is on clustering of items.

Second, the response rate of 38.4% for individual questionnaires is not very high, yet it is not unusual for an open population study. The low response could be due to the fact that because of the organisational structure, primary care professionals have no overhead time for such activities. On top of this, data collection was hindered because the membership records of some groups were not up to date. In addition, we were not able to distinguish the single-handed practices beforehand. Still, with a total of 615 questionnaires the rule of thumb of a ratio of ten respondents per item is amply met with a ratio of 14:1.

Third, a general drawback of measuring culture with a survey is that it will not capture the heart of the current culture, for which a more sophisticated method is necessary [23]. Options given are participant observation, interviews and focus groups combined with attitudinal surveys and established cultural assessment tools. Indeed, it would be interesting to combine the SCOPE-PC questionnaire with qualitative methods in a future study aiming at describing patient safety culture. However, for professionals themselves, as final users of the product, a survey has the advantage that it is feasible and easy to use.

## Conclusions

In conclusion, this study showed that the SCOPE-PC questionnaire for primary care has sound psychometric properties. The questionnaire is slightly different from the original SCOPE, but overall the main part of the factor structure is the same and only two items were deleted. In future, when sufficient data will be available, it would be interesting to perform cross-validation of the questionnaire. The use of the questionnaire will enable all professions in primary care to gain insight in their safety culture status and to take steps from there to improve patient safety in their practices. In our opinion, the next step in research is to explore the status and possible differences between professions in the Dutch primary care regarding patient safety culture.

## Competing interests

The authors declare that they have no competing interests.

## Authors’ contributions

DZ, ML, NV, TV and CW conceived and designed the study. NV carried out the collection, analyses and interpretation of the data and wrote the manuscript. DZ and ML participated in the interpretation of the analyses and co-wrote the manuscript. TV and CW revised the manuscript for important intellectual content. All authors read and approved the final manuscript.

## Pre-publication history

The pre-publication history for this paper can be accessed here:

http://www.biomedcentral.com/1472-6963/13/354/prepub

## Supplementary Material

Additional file 1 Description confirmatory factor analysis (CFA). Click here for file

Additional file 2 Scree plot. Click here for file
