# Supplementary material for: Measuring safety culture in Dutch primary care: psychometric characteristics of the SCOPE-PC questionnaire
Source: BMC Health Serv Res. 2013 Sep 17;13:354. doi: 10.1186/1472-6963-13-354 (PMC3851468; doi:10.1186/1472-6963-13-354)
Supplement: Additional file 1 — Description confirmatory factor analysis (CFA). [file 1472-6963-13-354-S1.pdf]

Additional file 1: Description confirmatory factor analysis (CFA)

A CFA provides a formal statistical test of how well the data fits the predisposed structure of factors. This fit is indicated by a χ^2 score and goodness-of-fit indices. The CFA was performed in Lisrel version 8.8. Because CFA was performed on a data file with missing data, Lisrel automatically uses Full Information Maximum Likelihood estimation. This method makes maximal use of all data available from every respondent in the sample. When performing a confirmatory factor analysis with missing values, Lisrel only gives χ^2 and the Root Mean Square Error of Approximation (RMSEA).

Interpretation of results

A non-significant χ^2 means that the discrepancies between the hypothesized model and the empirical data are negligible small and thus indicate a good fit. However, the χ^2 is sensitive to sample size and therefore little discrepancies can be statistical significant. The RMSEA measures how well the empirical model approaches the theoretical model. The assumption is that all models can only be an approximation and therefore a perfect fit cannot be obtained. A value of <0.05 is considered a close fit of the model, a value of <0.08 fair or a reasonable error of approximation, and values >0.1 are not acceptable.
