# Supplementary figures and images for: Measuring safety culture in Dutch primary care: psychometric characteristics of the SCOPE-PC questionnaire
Source: BMC Health Serv Res. 2013 Sep 17;13:354. doi: 10.1186/1472-6963-13-354 (PMC3851468; doi:10.1186/1472-6963-13-354)

## Additional file 2: Scree plot

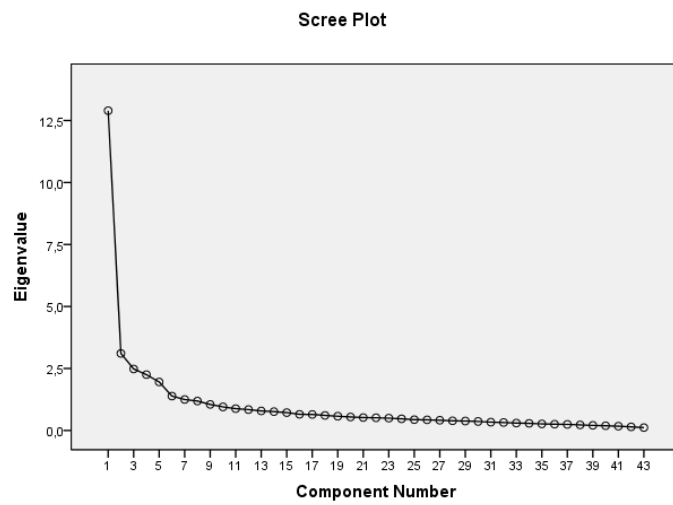

Supplement: Additional file 2 — Scree plot. [file 1472-6963-13-354-S2.pdf]
